# Complete Genome Sequences of *Aerococcus christensenii* CCUG 28831^T^, *Aerococcus sanguinicola* CCUG 43001^T^, *Aerococcus urinae* CCUG 36881^T^, *Aerococcus urinaeequi* CCUG 28094^T^, *Aerococcus urinaehominis* CCUG 42038 B^T^, and *Aerococcus viridans* CCUG 4311^T^

**DOI:** 10.1128/genomeA.00302-16

**Published:** 2016-04-21

**Authors:** Derya Carkaci, Rimtas Dargis, Xiaohui Chen Nielsen, Ole Skovgaard, Kurt Fuursted, Jens Jørgen Christensen

**Affiliations:** a Department of Clinical Microbiology, Slagelse Hospital, Slagelse, Denmark; b Department of Science and Environment, Roskilde University, Roskilde, Denmark; c Department of Microbiology and Infection Control, Statens Serum Institut, Copenhagen, Denmark; d Department of Clinical Medicine, University of Copenhagen, Copenhagen, Denmark

## Abstract

Strains belonging to the genus *Aerococcus* are causative agents of human and animal infections, including urogenital infections, bacteremia/septicemia, and infective endocarditis. This study reports the first fully closed and complete genome sequences of six type strains belonging to the genus *Aerococcus* using a combination of Illumina HiSeq and PacBio sequencing technologies.

## GENOME ANNOUNCEMENT

The genus *Aerococcus* encompasses eight species, which are Gram-positive cocci. For a long time, *Aerococcus viridans* (1953) (1) was the only known species. An additional seven species were described: *A. urinae* (1992) (2), *A. christensenii* (1999) (3), *A. urinaehominis* (2001) (4), *A. sanguinicola* (2001) (5), *A. urinaeequi* (2005) (6), *A. suis* (2007) (7), and *A. vaginalis* (2014) (8). *Aerococcus* spp. have been reported as human pathogens and associated with urogenital infections, bacteremia/septicemia, and infective endocarditis (1–5, 7–9). *A. suis* (from pig farms), *A. urinaeequi* (from horses and cattle), and *A. vaginalis* (from cow) have only been described as pathogens in animals (6–8).

In fact, little is known about *Aerococcus* pathogenicity and virulence mechanisms for causing infections in human and animals (10–13). One explanation for this has been stated as underestimated incidence in clinical settings, partly due to misidentification with closely related Gram-positive cocci (10, 14–16).

To date, six *Aerococcus* strains (*A. christensenii, A. urinae*, two *A. urinaeequi*, and two *A. viridans*) have been whole-genome sequenced and published in NCBI (National Center for Biotechnology Information), but these strains were either nontype strains or incomplete genome sequences.

Here, we announce the complete genome sequences of six *Aerococcus* type strains: *A. christensenii* CCUG 28831^T^, *A. sanguinicola* CCUG 43001^T^, *A. urinae* CCUG 36881^T^, *A. urinaeequi* CCUG 28094^T^, *A. urinaehominis* CCUG 42038 B^T^, and *A. viridans* CCUG 4311^T^.

The type strains were cultivated at 35 to 37°C for 9 to 10 h in Todd-Hewitt media with shaking in 5% CO_2_ atmosphere. Isolation and purification of high-quality genomic DNA was achieved using the Qiagen Genomic-tip 500/G system and the corresponding Genomic DNA buffer set with additional lysozyme and mutanolysin to improve bacterial cell lysis.

Illumina HiSeq (Illumina, USA) and PacBio (Pacific Biosciences, USA) sequence reads were generated at BGI (BGI, Shenzhen, China) and used in combination to obtain complete genome sequences. The HiSeq library sizes of 500-bp and 2-kb (500-bp and 6-kb for *A. urinaehominis* CCUG 42038 B^T^) were prepared using a BGI in-house method and were sequenced using the Illumina HiSeq2000 platform. The 20-kb PacBio Template Prep Kit was used to generate the PacBio libraries, followed by PacBio RS II sequencing. SOAPdenovo version 2.04 (17) was used to assemble preprosedded HiSeq sequence reads, whereas PacBio sequence reads were assembled using the Celera Assembler version 8.1 from the SMRT Analysis version 2.3.0 (http://www.pacb.com/products-and-services/analytical-software/smrt-analysis/). The combined HiSeq and PacBio assemblies generated one single contig of 1.6 to 2.2 Mb with 100% HiSeq coverages of the PacBio assemblies (Table 1). The NCBI Prokaryotic Genome Annotation pipeline version 3.1 (18) was used to annotate 1,428 to 1,914 coding genes, and plasmids were not detected in any of the genomes.

**TABLE 1:** *Aerococcus* type strain information, genome sequence data, and GenBank accession numbers

Type strain	Sequence depth		PacBio sequence statistic			NCBI prokaryotic genome annotation pipeline			General strain information			Accession no.^a^
	PacBio	HiSeq	No. of contigs	Genome size (bp) and *N*_50_ (bp)	G+C content (%)	No. of coding genes	No. of 5S, 16S, and 23S rRNAs	No. of tRNAs	Yr of strain announcement	Source of isolation	Host disease	
*A. christensenii* CCUG 28831^T^	467×	290×	1	1,634,920	39.2	1,428	4, 4, 4	60	1988/1999^b^	Human vagina	Vaginosis	CP014159
*A. sanguinicola* CCUG 43001^T^	515×	210×	1	2,033,849	47.6	1,733	4, 4, 4	62	2001	Human blood	Septicemia	CP014160
*A. urinae* CCUG 36881^T^	594×	190×	1	1,974,262	42.6	1,712	4, 4, 4	60	1989/1992^c^	Human urine	Urinary tract infection	CP014161
*A. urinaeequi* CCUG 28094^T^	348×	220×	1	2,013,339	39.4	1,761	5, 5, 5	54	1934/1988/2005^d^	Horse urine	ND^e^	CP014162
*A. urinaehominis* CCUG 42038 B^T^	592×	170×	1	1,831,400	42.8	1,605	4, 4, 4	57	2001	Human urine	Urinary tract infection	CP014163
*A. viridans* CCUG 4311^T^	486×	250×	1	2,199,877	39.4	1,914	7, 7, 7	55	1953	Air sample	ND	CP014164

^a^ Bioproject accession number PRJNA308559.

^b^ Reclassification of *Streptococcus acidominimus* (1988) as *Aerococcus christensenii* in 1999.

^c^ Characterization of *Aerococcus*-like organisms (1989) as *Aerococcus urinae* in 1992.

^d^ Reclassification of *Pediococcus urinaeequi* ([ex Mees 1934] Garvie 1988) as *Aerococcus urinaeequi* in 2005.

^e^ ND, not defined.

Genome sequences were in silico NCBI BLAST (19) confirmed by rRNAmmer version 1.2 (20) predicted 16S rRNA gene sequences achieving 99 to 100% sequence identities with corresponding NCBI deposited 16S rRNA gene sequences. Additional analysis of 16S-23S rRNA gene sequences distinguished *A. viridans* from *A. urinaeequi*.

The availability of these six *Aerococcus* type strain complete genome sequences will provide important information concerning the genetic content of the genus *Aerococcus*. These genomes will act as reference strains in terms of comparative genomics in relation to pathogenicity, which will improve the understanding of *Aerococcus*-associated infections in the future.

### Nucleotide sequence accession numbers.

The complete genome sequences of the six *Aerococcus* type strains were deposited in GenBank under the accession numbers stated in Table 1.
